# Malaria prevalence in Mauritania: a systematic review and meta-analysis

**DOI:** 10.1186/s12936-023-04569-4

**Published:** 2023-05-02

**Authors:** Inejih El Moustapha, Mohamed Ouldabdallahi Moukah, Mohamed Salem Ould Ahmedou Salem, Khyarhoum Brahim, Sébastien Briolant, Leonardo Basco, Ali Ould Mohamed Salem Boukhary

**Affiliations:** 1 Université de Nouakchott, UR-GEMI, nouveau campus universitaire, BP 5026 Nouakchott, Mauritania; 2 Aix Marseille Université, IRD, AP-HM, SSA, VITROME, Marseille, France; 3 IHU-Méditerranée Infection, Marseille, France; 4 Unité de Parasitologie Entomologie, Département de Microbiologie et Maladies Infectieuses, Institut de Recherche Biomédicale des Armées (IRBA), Marseille, France

**Keywords:** Malaria, Meta-analysis, Mauritania, Prevalence, *Plasmodium falciparum*, *Plasmodium vivax*, Systematic review

## Abstract

**Background:**

Understanding malaria epidemiology is a critical step toward efficient malaria control and elimination. The objective of this meta-analysis was to derive robust estimates of malaria prevalence and *Plasmodium* species from studies conducted in Mauritania and published since 2000.

**Methods:**

The present review followed the PRISMA guidelines. Searches were conducted in various electronic databases such as PubMed, Web of Science, and Scopus. To obtain pooled prevalence of malaria, meta-analysis was performed using the DerSimonian-Laird random-effects model. Methodological quality of eligible prevalence studies was assessed using Joanna Briggs Institute tool. Inconsistency and heterogeneity between studies were quantified by the I^2^ index and Cochran’s Q test. Publication bias was assessed with funnel plots and Egger’s regression tests.

**Results:**

A total of 16 studies with a good individual methodological quality were included and analysed in this study. The overall random effects pooled prevalence of malaria infection (symptomatic and asymptomatic) across all included studies was 14.9% (95% confidence interval [95% CI]: 6.64, 25.80, I^2^ = 99.8%, P < 0.0001) by microscopy, 25.6% (95% CI: 8.74, 47.62, I^2^ = 99.6%, P < 0.0001) by PCR and 24.3% (95% CI: 12.05 to 39.14, I^2^ = 99.7%, P < 0.0001) by rapid diagnostic test. Using microscopy, the prevalence of asymptomatic malaria was 1.0% (95% CI: 0.00, 3.48) against 21.46% (95% CI: 11.03, 34.21) in symptomatic malaria. The overall prevalence of *Plasmodium falciparum* and *Plasmodium vivax* was 51.14% and 37.55%, respectively. Subgroup analysis showed significant variation (P = 0.039) in the prevalence of malaria between asymptomatic and symptomatic cases.

**Conclusion:**

*Plasmodium falciparum* and *P. vivax* are widespread in Mauritania. Results of this meta-analysis implies that distinct intervention measures including accurate parasite-based diagnosis and appropriate treatment of confirmed malaria cases are critical for a successful malaria control and elimination programme in Mauritania.

**Supplementary Information:**

The online version contains supplementary material available at 10.1186/s12936-023-04569-4.

## Background

In Mauritania, malaria is endemic with a seasonal and unstable transmission [1–4]. The transmission peaks in October and November, coinciding with the end of the short rainy season which extends from July to September. The presence of four human malaria species (i.e., *Plasmodium falciparum*, *Plasmodium vivax*, *Plasmodium ovale*, and *Plasmodium malariae*) has been reported in Mauritania [1–6]. *Plasmodium falciparum* and *P. vivax* are by far the most predominant malaria species in the country [2, 3, 6]. In the Sahelian zone where *P. falciparum* prevails, *P. vivax* is rare [3]. Inversely, *P. vivax* is the major malaria species in northern Saharan arid zone, notably in oases [2, 6]. However, the factors that govern the geographical distribution of *P. falciparum* and *P. vivax* across the two ecological zones in Mauritania, i.e. Sahelian and Saharan zones, respectively, are still unknown. *Anopheles arabiensis* is widespread in the country [7–12]. It is the principal malaria vector in Mauritania, with the possible exception of the far northern Saharan regions (e.g. Atar) where entomological surveys have been limited.

Malaria diagnosis is generally established in health facilities either by presumptive clinical diagnosis or, more often, by *P. falciparum*-specific histidine-rich protein-2 (HRP2)- and *Plasmodium* genus-specific plasmodial lactate dehydrogenase (‘Pan’ pLDH)-based rapid diagnostic test (RDT). Studies on the performance of some RDTs for malaria reported contrasting levels of sensitivity and specificity to detect malaria [3, 13]. Microscopy, the gold standard for malaria diagnosis in the field according to the World Health Organization (WHO) [14], is rarely performed to confirm malaria infections outside of clinical research, and the use of polymerase chain reaction (PCR) assays, which are more sensitive to detect malaria parasites, remains limited to research purposes.

The main target of the current malaria control strategy of the Mauritanian government is to eliminate malaria from the country by 2030 [15]. This strategy includes prompt diagnosis and appropriate treatment of laboratory-confirmed malaria cases with artemisinin-based combination therapy (ACT; i.e. artesunate-amodiaquine or artemether-lumefantrine), regardless of *Plasmodium* species, mass distribution of long-lasting insecticidal nets (LLIN), intermittent preventive treatment in pregnant women using sulfadoxine-pyrimethamine (IPTp-SP), and more recently, seasonal malaria chemoprevention (SMC) in children under five years of age [16, 17]. However, to achieve malaria elimination, it is critical to understand the epidemiology of malaria infection.

During the past two decades, numerous studies on malaria prevalence have been conducted in Mauritania by different investigators using various approaches in different ecological conditions and epidemiological context (variable sample size and sampling technique, study sites, period of data collection, and characteristics of study participants), resulting in heterogeneous malaria positivity rates and *Plasmodium* species distribution [1–3, 5, 6, 13, 18–27]. To obtain a weighted average of malaria prevalence from such studies, meta-analysis has been demonstrated as a valuable and efficient approach [28, 29]. The objectives of this study were to provide synthetic data from malaria prevalence studies published since 2000 in Mauritania and derive robust estimates of malaria positivity rates and proportions of different malaria species in the country. A clear understanding of malaria prevalence and *Plasmodium* species distribution is essential for informed decisions on appropriate control strategies.

## Methods

### Study area

**Fig. 1:**
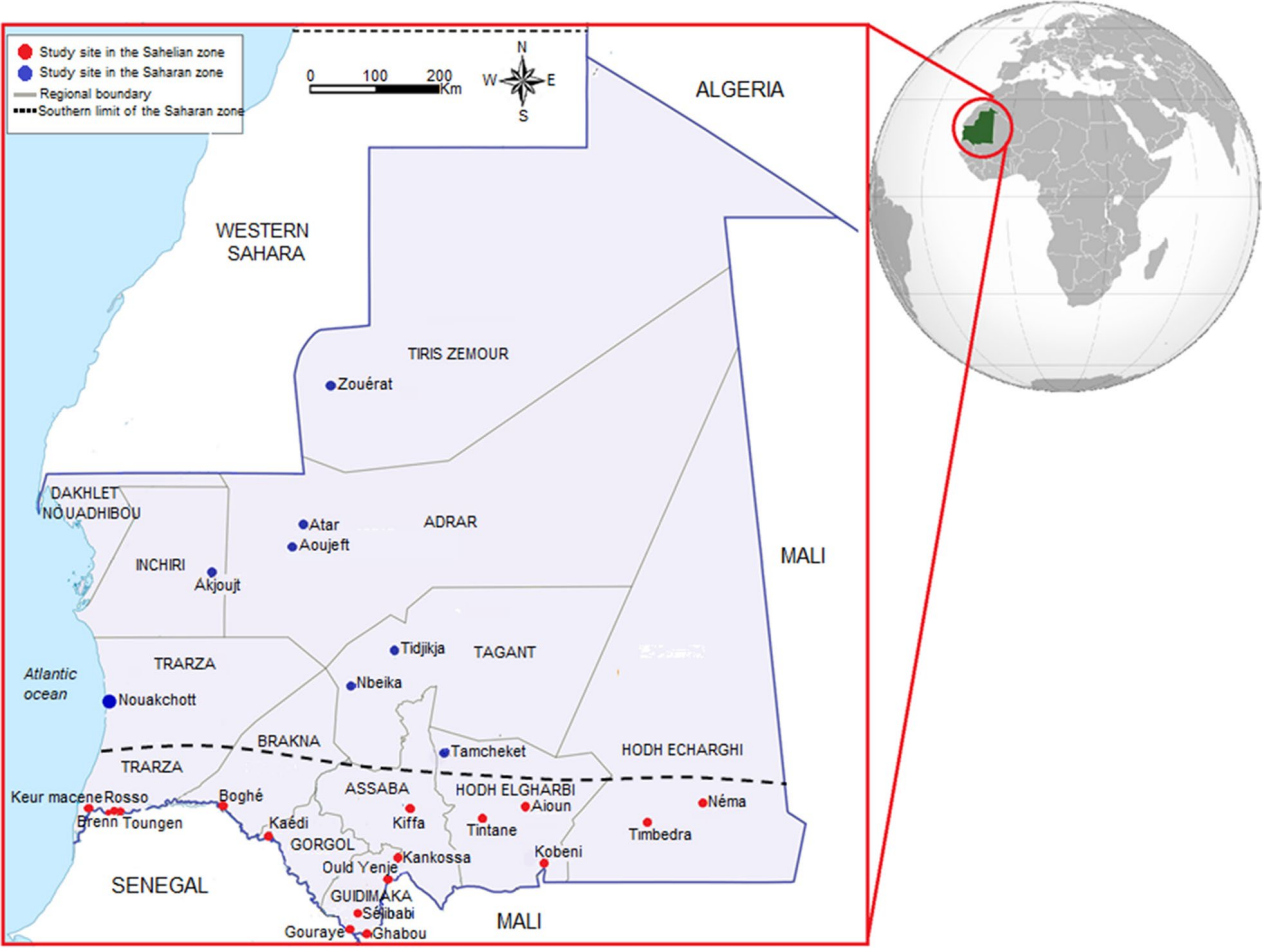
Map of Mauritania and study sites. Geographical distribution of locations from which malaria prevalence was reported is shown. Red circles, sites in the Sahelian zone; blue circles, sites in the Saharan zone

Mauritania is a vast country located at the crossroads of the Maghreb region (north African region that includes Morocco, Algeria and Tunisia) and sub-Saharan Africa with a surface area of 1,080,000 km^2^ (Fig. 1). It lies between 15 and 27° north latitude and 5 and 17° west longitude and is bordered by Senegal to the south west, Mali to the east and south east, Algeria to the north east, Western Sahara to the north west and the Atlantic Ocean to the west.

The total estimated population was 4.7 million in 2020, of whom approximately one fourth are living in Nouakchott, the capital city [30]. In the northern two-thirds of the country, the climate is Saharan with scarce rainfall (< 200 mm annually), while in the southern one-third of the remaining zone, the climate is Sahelian with an annual rainfall between 200 and 500 mm. Mauritania has a single rainy season from July to September in the south with shorter duration of the rainy season and decreasing amount of precipitation towards the north [31]. Mean annual temperatures range from 21° to 30 °C, with relatively lower temperature along the northern coast and higher temperature in the southeastern region of the country [31]. According to the WHO, 80% of the Mauritanian population live in malaria-prone areas [32].

### Searching strategy

The literature search strategy, selection of publications, data extraction, and the reporting of results for the review were performed following the Preferred Reporting Items for Systematic Reviews and Meta-Analyses (PRISMA) guidelines [33]. Published articles were identified in PubMed, Web of Science, and Scopus databases, as well as in the institutional database of the French National Research Institute for Sustainable Development (Institut de Recherche pour le Développement [IRD]) (https://www.ird.fr/bureau-du-chercheur) for papers published in French. The database search was performed using a combination of terms/keywords and Boolean operators according to each database instructions (see Additional file 1: Table S1 for the full detailed search strategies).

### Inclusion and exclusion criteria

Studies published between January 2000 and January 2023 (the last search was performed on 20^th^ January 2023) in either English or French were included in the analysis. The inclusion criteria were original study conducted in Mauritania to assess malaria prevalence with basic information concerning the sample size, diagnostic methods, *Plasmodium* species, and the main characteristics of the enrolled subject. The exclusion criteria were as follows: studies published before January 1, 2000, reviews, letters to editors, editorials, commentaries, expert opinions, books, book chapters, brief reports, articles in which only the abstract was available, conference abstracts, theses, case reports, and case series. In addition, articles in which malaria prevalence was not determined and studies with reported malaria prevalence based on small (i.e., n < 30) sample size were also excluded.

### Quality assessment of individual studies

The methodological quality of the included studies was assessed using the Joanna Briggs Institute (JBI) quality assessment tool for prevalence studies [34]. The tool consists of nine questions designed to assess the extent to which a study has addressed the possibility of bias in its design, conduct and analysis. Studies with a total score of ≥ 50% were considered as having a low risk of bias. Two critical appraisers independently assessed methodological quality of the individual article.

### Data analysis

A quantitative pooling of results was done to perform a meta-analysis using MedCalc^®^ Statistical Software version 20.115 (MedCalc Software Ltd, Ostend, Belgium; https://www.medcalc.org; 2022). Cochrane Q test and Inconsistency index (denoted I^2^ statistics) were used to evaluate the magnitude of heterogeneity among studies included in meta-analysis. A Cochrane Q value with *P* < 0.05 indicated significant heterogeneity among studies. I^2^ values < 25%, 25–75% and > 75% were interpreted as low, moderate, and considerable heterogeneity, respectively.

Preliminary analysis showed a high degree of heterogeneity among the included studies (I^2^ = 99.5%, *P* < 0.001). As a result, a random-effects model was performed to estimate the Der Simonian and Laird's pooled effect. Subgroup analyses were conducted to explore the potential sources of heterogeneity, including the following variables: size of the study population, eco-epidemiological zone of the study settings, malaria diagnostic method used, status of study participants with respect to malaria (whether symptomatic or asymptomatic). Forest plots were used to display point estimates and confidence intervals.

Publication bias for studies included in the meta-analysis was assessed qualitatively by visual inspection of funnel plots and evaluated for asymmetry, and quantitatively using Egger’s regression test and Begg’s correlation test. A significance level *P* > 0.05 indicates no evidence of publication bias.

## Results

### Study selection and quality assessment of individual articles

**Fig. 2:**
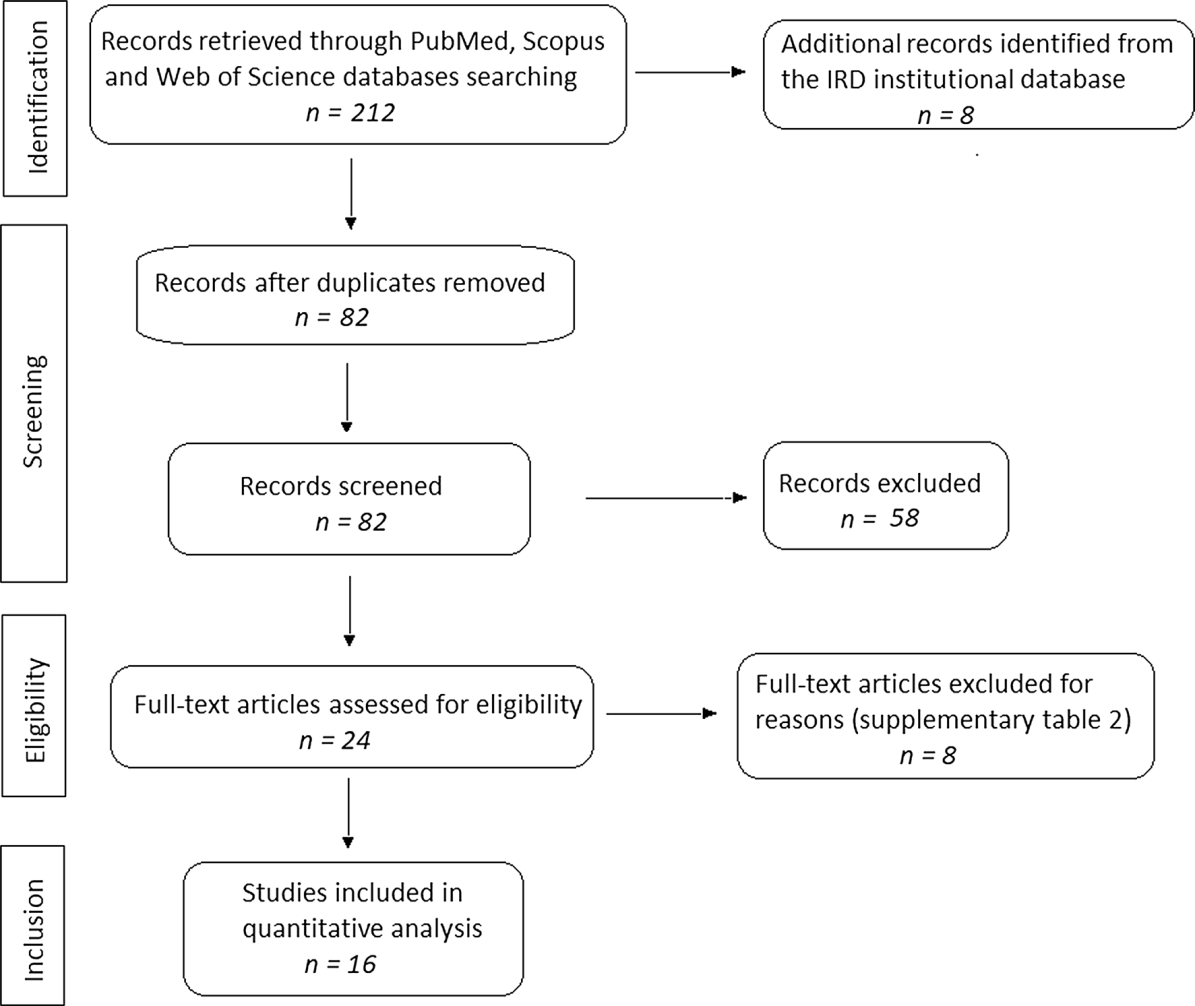
Flow chart of the literature search and selection process

A total of 220 records were retrieved from different databases. After duplicates were excluded, 82 records were screened and assessed at title/abstract level for eligibility (Fig. 2). From the remaining 82 records, 58 which were considered irrelevant for the purposes of the study were excluded. As a result, 24 eligible full-text articles were rigorously reviewed, and 8 additional articles were excluded. Detailed reasons for the excluded articles are given in Additional file 2: Table S2. The remaining 16 full-text papers (2 in French and 14 in English) [1–3, 5, 6, 11, 13, 18–26] met the inclusion criteria and were retained for the present systematic meta-analysis. All 16 articles that were analysed in this study satisfied the quality assessing criteria of the JBI guideline for prevalence studies for at least 7 of 9 domains, which demonstrated good methodological quality.

### Study sites and study period

Overall, studies published in 16 articles included 25 study sites (17 in the southern Sahelian zone and 8 in the northern Saharan zone) (Fig. 1). These study sites ranged from small settings in rural areas (e.g., two villages called Toungen and Brenn situated close to the city of Rosso along the Senegal River), to large urban settings such as Nouakchott, the capital city, Kiffa, and Kaedi. The study sites were sparsely distributed across the country with Gouraye in Guidimagha region bordering Mali, representing the southernmost study site, and Zouerat in the extreme north confine of the Sahara desert, the northernmost study site. The eastern half of the country was not represented in any of the studies because there is no human settlement in this vast desertic zone.

### Basic characteristics of the included studies

Tables 1 and 2 show the basic characteristics of the studies. Studies were performed in the field between 1996 and 2020, with the first survey carried out in 1996 and 1999 in Nouakchott and Kaedi, respectively [18] (Table 1). The last included article was a six-year prospective study (2015–2020) conducted in Nouakchott [6]. Of the 16 included studies that reported malaria prevalence, 15 (93.7%) were cross-sectional, including 1 (6.3%) that assessed the performance of a RDT for malaria [13]. Another work (n = 1; 6.3%) was a retrospective study based on patient data collected from one heath facility in Nouakchott [23]. Of 15 cross-sectional studies, 12 (80%) reported data collected in health facilities, 2 (13.3%) reported data at the community level (i.e., households and schools), and 1 (6.7%) reported data from both health facilities and community level. In the majority of studies (12/16; 75%), surveys were carried out over several years and during both rainy and dry seasons. Data on malaria prevalence were obtained from only one study site in 9 (56.2%) studies and from 2 to 12 (12, 11, 9, 6, 4, 3, and 2) study sites in the remaining 7 studies.

**Table 1:** Basic characteristics of studies reporting malaria prevalence in Mauritania since 2000

Author(s)	Study year	Season	Study design	Setting	Study site (Bioclimatic zone)
Cortes et al. (2003)	1996	Dry and wet seasons	Cross-sectional	Health facilities	Nouakchott (Sahara)
	1999				Kaedi (Sahel)
Ouldabdallahi et al. (2011)	2004–2006	Dry and wet seasons	Cross-sectional	Health facilities,	Rosso (Sahel)
			Cross-sectional	Schools	Toungen, Brenn (Sahel)
Mint Lekweiry et al. (2009)	2007	Dry season	Cross-sectional	Health facilities	Nouakchott (Sahara)
Mint Lekweiry et al. (2011)	2009–2010	Dry season	Cross-sectional	Health facilities	Nouakchott (Sahara)
Ould Ahmedou Salem et al. (2015a)	2009–2010	Wet season	Cross-sectional	Health facilities	Nouakchott (Sahara)
					Kobeni, Tintane, Aioun (Sahel)
Ouldabdallahi et al. (2015)	2010–2011	Dry and wet seasons	Cross-sectional	Health facilities	Keur macene, Boghé, Selibaby, Kankossa, Kobeni (Sahel)
					Akjoujt, Atar, Nouakchott, Tamcheket (Sahara)
Touray et al. (2012)	2011	Dry season	Cross-sectional	Households	Kaedi (Sahel)
Ouldabdallahi et al. (2016)	2011, 2012, 2013	Dry and wet seasons	Cross-sectional	Households	Keur macene, Boghé, Gouraye, Ghabou, Kobeni, Kankossa (Sahel)
					Tamcheket, Nouakchott, Aoujeft, Akjoujt, Nbeika (Sahara)
Ould Ahmedou Salem et al. (2015b)	2012–2013	Dry and wet seasons	Retrospective	Health facilities	Nouakchott (Sahara)
Ba et al. (2016)	2012–2013	Dry and wet seasons	Cross-sectional	Health facilities	Nouakchott, Zouerat, Nbeika, Tidjikja (Sahara)
					Kiffa, Boghe, Nema, Ould Yenje, Sélibaby, Timbedra, Kobeni, Aïoun (Sahel)
Ba et al. (2017)	2012–2013	Dry and wet seasons	Cross-sectional	Health facilities	Nouakchott, Nbeika (Sahara)
					Timbedra, Kobeni, Boghé, Aïoun (Sahara)
Gbalégba et al. (2018)	2014, 2015	dry and wet seasons	Cross-sectional	Households	Kaedi (Sahel)
Mint Deida et al. (2019)	2015–2016	Dry and wet seasons	Cross-sectional	Health facilities	Atar (Sahara)
Ould Lemrabott et al. (2021)	2015–2016	Dry and wet seasons	Cross-sectional	Health facilities	Rosso (Sahel)
Diallo et al. (2020)	2015–2017	Dry and wet seasons	Cross-sectional	Health facilities	Kobeni (Sahel)
El Moustapha et al. (2023)	2015–2020	Dry and wet seasons	Cross-sectional	Health facilities	Nouakchott (Sahara)

**Table 2:** Study population characteristics, malaria diagnostic method and *Plasmodium* species reported in Mauritania since 2000

Author(s)	Characteristics of the study population				Method of Diagnosis	Malaria positive using microscopy						Malaria positive using PCR			
	*N*	Type of cases	Age (year)	Sex ratio		Total	*Pf*	*Pv*	*Po*	*Pm*	Mixed	Total	*Pf*	*Pv*	Mixed
Cortes et al. (2003)	862	Malaria suspected	All ages	NG	M	183	153	28	0	0	0	NA	NA	NA	NA
Mint Lekweiry et al. (2009)	237	Malaria suspected	All ages	1.4	M, P	61	1	43	15	0	2 [*Pv-Po*]	59	1	58	0
Ouldabdallahi et al. (2011)	1431	Malaria suspected	All ages	0.9	M	36	29	7	0	0	0	NA	NA	NA	NA
	1040	Asymptomatic	6–14	1.1		9	8	0	0	1	0				
Mint Lekweiry et al. (2011)	301	Malaria suspected	0–14	1.3	R, M, P	87	3	84	0	0	0	105	3	102	0
Touray et al. (2012)	371	Asymptomatic	1–5	1.1	R, M	0	0	0	0	0	0	NA	NA	NA	NA
Ould Ahmedou Salem et al. (2015a)	1161	Malaria suspected	All ages	1.1	R, M	631	408	213	2	4	4 [*Pf-Pv*]	NA	NA	NA	NA
Ould Ahmedou Salem et al. (2015b)	9141	Malaria suspected	All ages	NG	R	NA	NA	NA	NA	NA	NA	NA	NA	NA	NA
Ouldabdallahi et al. (2015)	7368	Malaria suspected	All ages	0.8	M	672	218	412	34	5	3	NA	NA	NA	NA
Ouldabdallahi et al. (2016)	3445	Asymptomatic	2–9	1.0	R, M	143	71	59	11	2	0	NA	NA	NA	NA
Ba et al. (2016)	472	Malaria positive	All ages	NG	R, P	NA	NA	NA	NA	NA	NA	472	296	163	13 [*Pf-Pv*]
Ba et al. (2017)	759	Malaria suspected	All ages	1.1	R, M	200	127	59	0	0	14	NA	NA	NA	NA
Gbalégba et al. (2018)	9165	Asymptomatic	All ages	0.6	R, M	23^a^	13	1	0	2	0	NA	NA	NA	NA
Deida et al. (2019)	453	Malaria suspected	All ages	1.2	R, M, P	154	4	140	10	0	0	162	4	120	38 [*Pf*-*Pv*]
Diallo et al. (2020)	2326	Malaria suspected	All ages	0.9	R, M, P	1146	1143	0	2	1	0	1361^b^	1205	47	99 [*Pf-Pv*] 6 [*Pf-Pm*] 1 [*Pf-Pv-Pm*]
Ould Lemrabott et al. (2021)	318	Malaria suspected	All ages	0.9	R, M, P	2	2	0	0	0	0	2	0	2	0
El Moustapha et al. (2023)	1760	Malaria suspected	All ages	0.9	R, M, P	256	NG	NG	NG	NG	NG	291	47	216	28 [*Pf-Pv*]

*Pf*: *Plasmodium falciparum*; *Pv*: *Plasmodium vivax*; *Po*: *Plasmodium ovale*; *Pm*: *Plasmodium malariae*; NA: not applicable; NG: not given; R, rapid diagnostic test for malaria; M: microscopy; P: polymerase chain reaction

^a^*Plasmodium* spp. infections could not be identified in 7 positive cases due to the deterioration of the thin blood films

^b^Including 3 *P. malariae* mono-infection by PCR [3]

A total of 40,138 subjects, ranging from 237 [19] to 9,165 [26], were enrolled in 15 of 16 included studies (Table 2). In the remaining study, only the number of malaria-positive subjects was given as the objective of that study was to compare the prevalence of *Plasmodium* species among malaria-positive patients [5].

Of 40,138 subject enrolled, 22,034 (54.9%) were from the southern Sahelian region and 18,104 (45.1%) from the northern Saharan region (*P* < 0.0001). The majority of the subjects (37%; 14,870/40,138) were enrolled in Nouakchott. Participants in 11 of 16 (68.7%) included studies were febrile patients with suspected malaria recruited at health facilities, while they were asymptomatic in 3 (18.8%) studies, both asymptomatic and febrile subjects in 1 (6.2%) study, and only malaria-positive patients in 1 (6.2%) study (Table 2). Participants in 3 of 4 studies included asymptomatic individuals screened for malaria among children under 14 years of age.

Enrolled participants belonged to all age groups in 13 of 16 (81%) included studies and children under 14 years in 3 (18.8%) studies. The sex of the participants was reported in 13 of 16 (81.2%) studies. Overall, there was significant difference (*P* = 0.0018) between the proportions of males (50.9%; 15,339/30,135) and females (49.1%; 14,796/30,135).

Malaria diagnostic methods were based on three techniques (RDT, microscopy, and PCR) in 5 (31.2%) studies, and both RDT and microscopy in 6 (37.5%) other studies. In the remaining 5 (31.2%) studies, malaria diagnosis was based on microscopy alone (n = 3), microscopy and PCR (n = 1), or RDT alone (n = 1).

With microscopy, four human malaria species (i.e. *P. falciparum*, *P. vivax*, *P. malariae,* and *P. ovale*) were diagnosed among enrolled patients with a majority of cases (n = 2,180) being infected by *P. falciparum*, followed by those infected by *P. vivax* (n = 1,046). Rare cases of *P. falciparum*-*P. vivax* mixed infections (n = 4) and *P. vivax*-*P. ovale* (n = 2) were also recorded (Table 2).

In studies in which PCR was employed to confirm diagnosis, only *P. falciparum*, *P. vivax,* and *P. malariae* were detected, either as mono-infection or mixed infections, with a total of 2,452 PCR-positive cases [2, 3, 5, 6, 19, 20]. Mono-infections included 1,556 *P. falciparum* cases, 708 *P. vivax* cases, and 3 *P. malariae* cases. Mixed infections (n = 185) consisted of 178 *P. falciparum-P. vivax* mixed infections, 6 *P. falciparum*-*P. malariae* mixed infections, and 1 *P. falciparum*-*P. vivax*-*P. malariae* mixed infection (Table 2) [2, 3, 5, 6].

### Main meta-analysis outcome

**Fig. 3:**
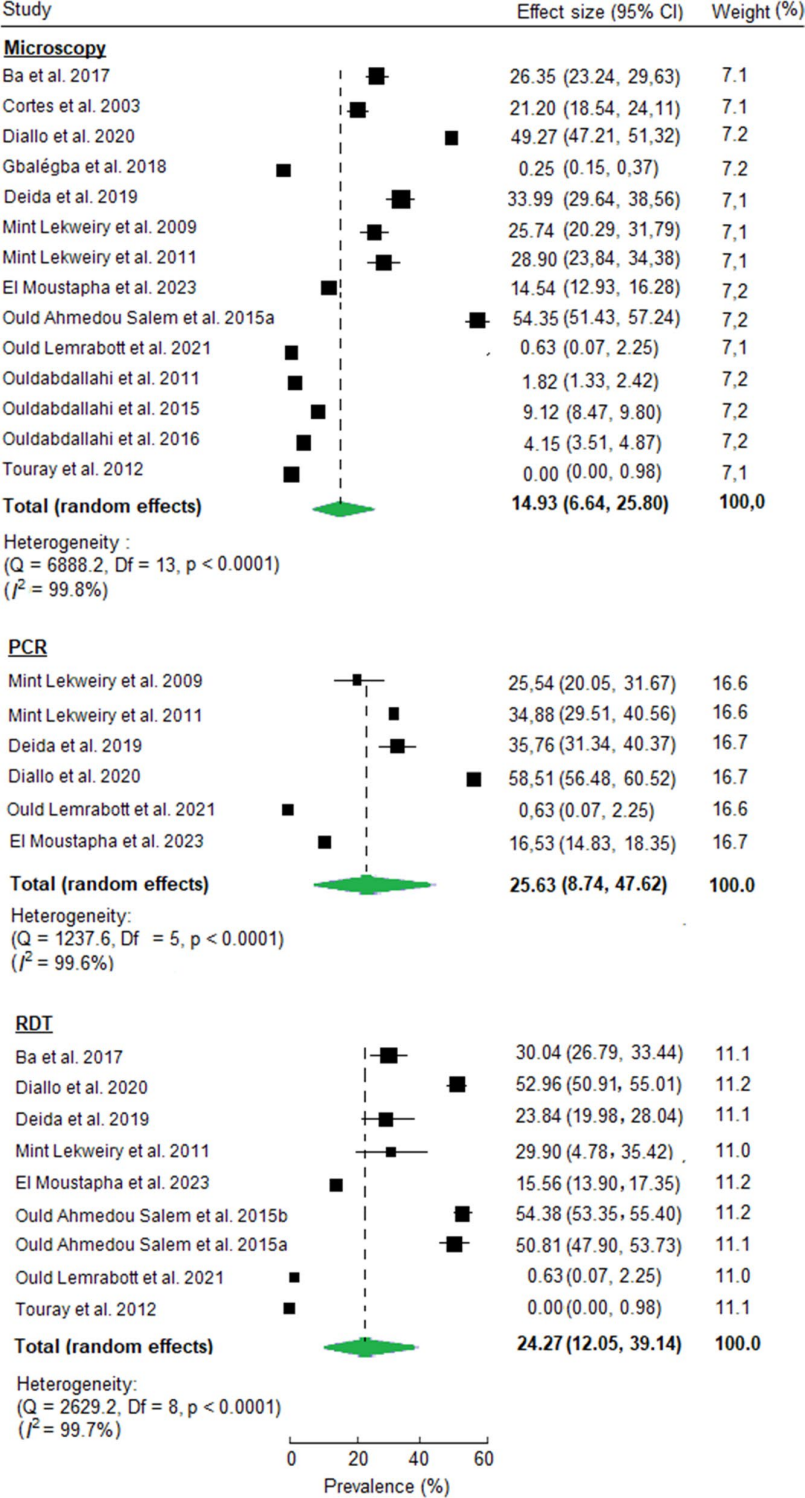
Forest plots showing random effects of individual and pooled estimates of malaria prevalence in Mauritania by diagnostic method

The overall random effects pooled prevalence of malaria infection across all included studies was 14.9% (95% CI: 6.64, 25.80, I^2^ = 99.8%, *P* < 0.0001) by microscopy (14 studies and 30,997 subjects), 25.6% (95% CI: 8.74, 47.62, I^2^ = 99.6%, *P* < 0.0001) by PCR (6 studies and 5,389 subjects), and 24.3% (95% CI: 12.05 to 39.14, I^2^ = 99.7%, *P* < 0.0001) by RDT (9 studies and 16,590 subjects) (Fig. 3).

According to microscopy, the gold standard for laboratory confirmation of malaria, the minimum prevalence of malaria was 0.25% (95% CI: 0.15, 0.37) observed in asymptomatic individuals in Kaedi situated along the Senegal River [26], while the maximum prevalence of malaria parasites was 54.4% (95% CI: 51.43, 57.24), observed among febrile patients consulting at health facility in Hodh El Gharbi province (southeastern Mauritania) [1].

**Fig. 4:**
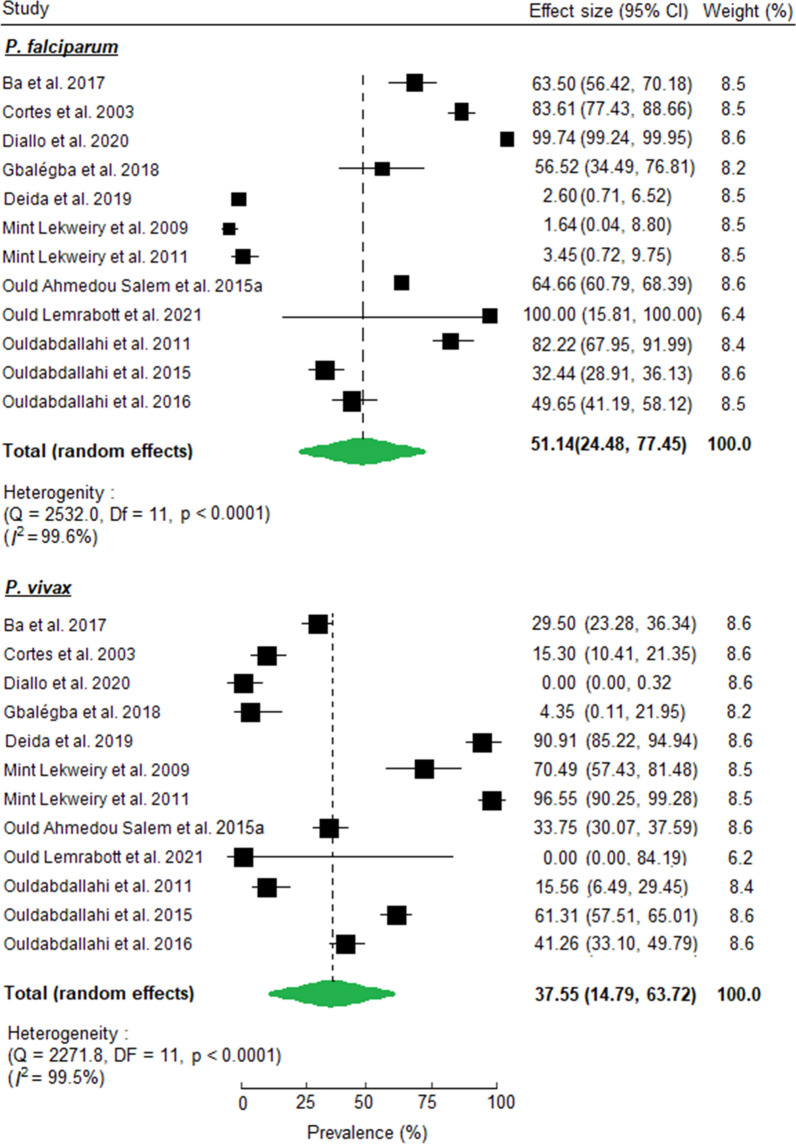
Forest plots showing individual and pooled estimates of *P. falciparum* and *P. vivax* prevalence using microscopy in Mauritania

*Plasmodium falciparum* and *P. vivax* were the two dominant species using microscopy (n = 12 studies) with a pooled prevalence of 51.1% (95% CI: 24.5, 77.4) and 37.5% (95% CI: 14.8, 63.7), respectively (Fig. 4). To assess the prevalence of mixed infections, PCR results showed that *P. falciparum*-*P. vivax* mixed infection accounted for 7.5% (178/2,452) of the global malaria infections and 96.2% (178/185) of the mixed infections.

### Sub-group analysis

**Fig. 5:**
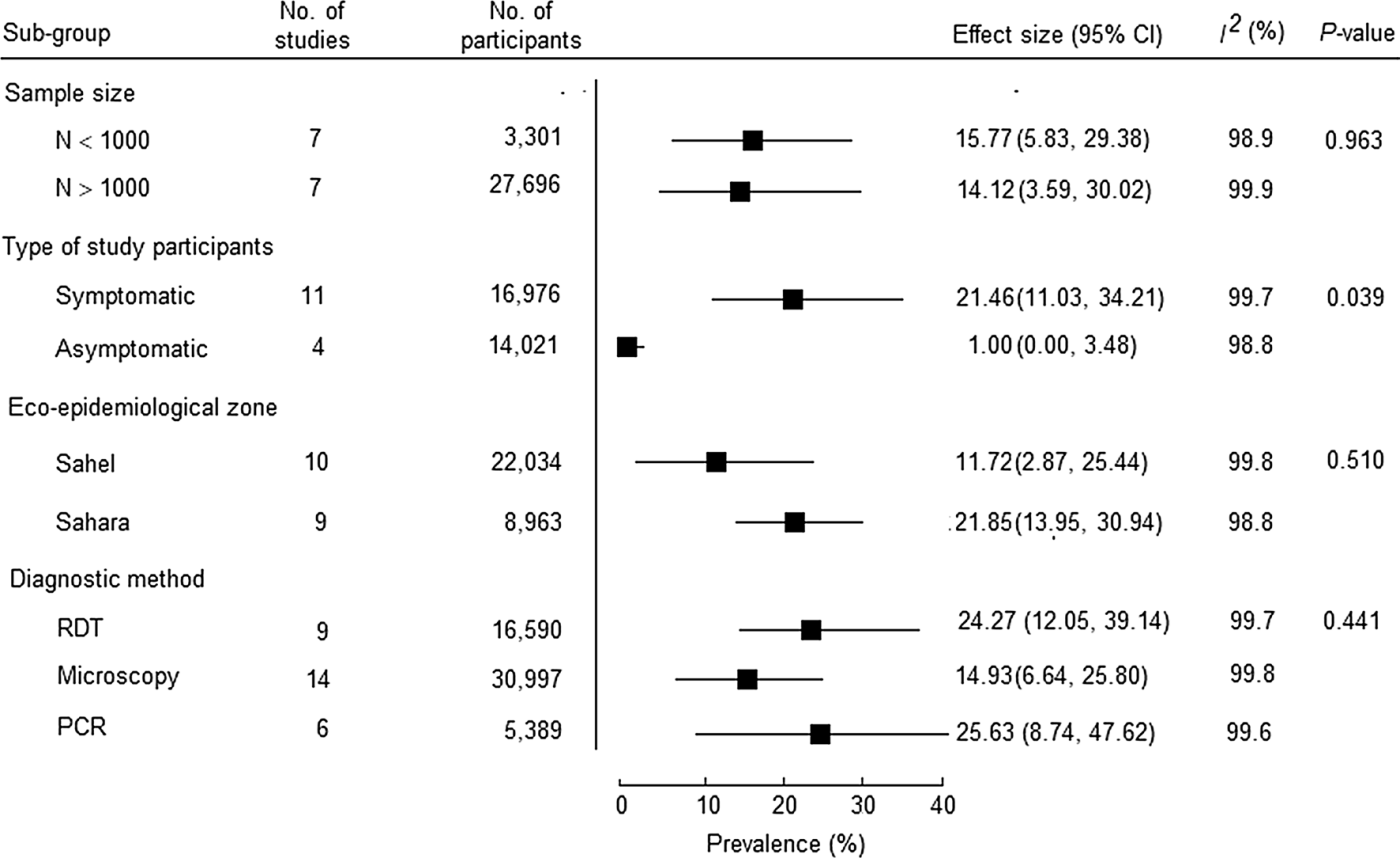
Subgroup analysis of the possible source of heterogeneity among individual studies using microscopy in Mauritania

Due to the highly significant heterogeneity among included studies (i.e. I^2^ > 97% in all subgroups, *P* < 0.0001), sub-group meta-analysis was performed by eco-epidemiological zones of the study sites (i.e. Sahel vs. Sahara), sample size of enrolled subjects by study (N < 1,000 enrolled subject vs. N > 1,000 enrolled subjects), malaria diagnostic method (microscopy vs RDT vs PCR), and type of malaria cases (i.e. symptomatic vs. asymptomatic) (Fig. 5).

Accordingly, the meta-analysis showed no significant difference (*P* = 0.963) in malaria prevalence between studies having sample size > 1,000 study participants (14.1%, 95% CI: 3.59, 30.02) and those having sample size < 1,000 study participants (15.8%; 95% CI: 5.8, 29.4). However, a significant difference (*P* = 0.039) was observed between malaria prevalence in symptomatic patients (21.5%; 95% CI: 11.0, 34.2) compared to asymptomatic individuals (1%; 95% CI: 0, 3.5). The estimated prevalence of malaria in the Sahelian zone was 11.7% (95% CI: 2.87, 25.44), while the estimated malaria prevalence in the Saharan zone was 21.8 (95% CI: 14.0, 30.9) (*P* = 0.510). There was no significant difference between malaria prevalence according to the diagnostic method employed (*P* = 0.441).

### Assessment of publication bias

**Fig. 6:**
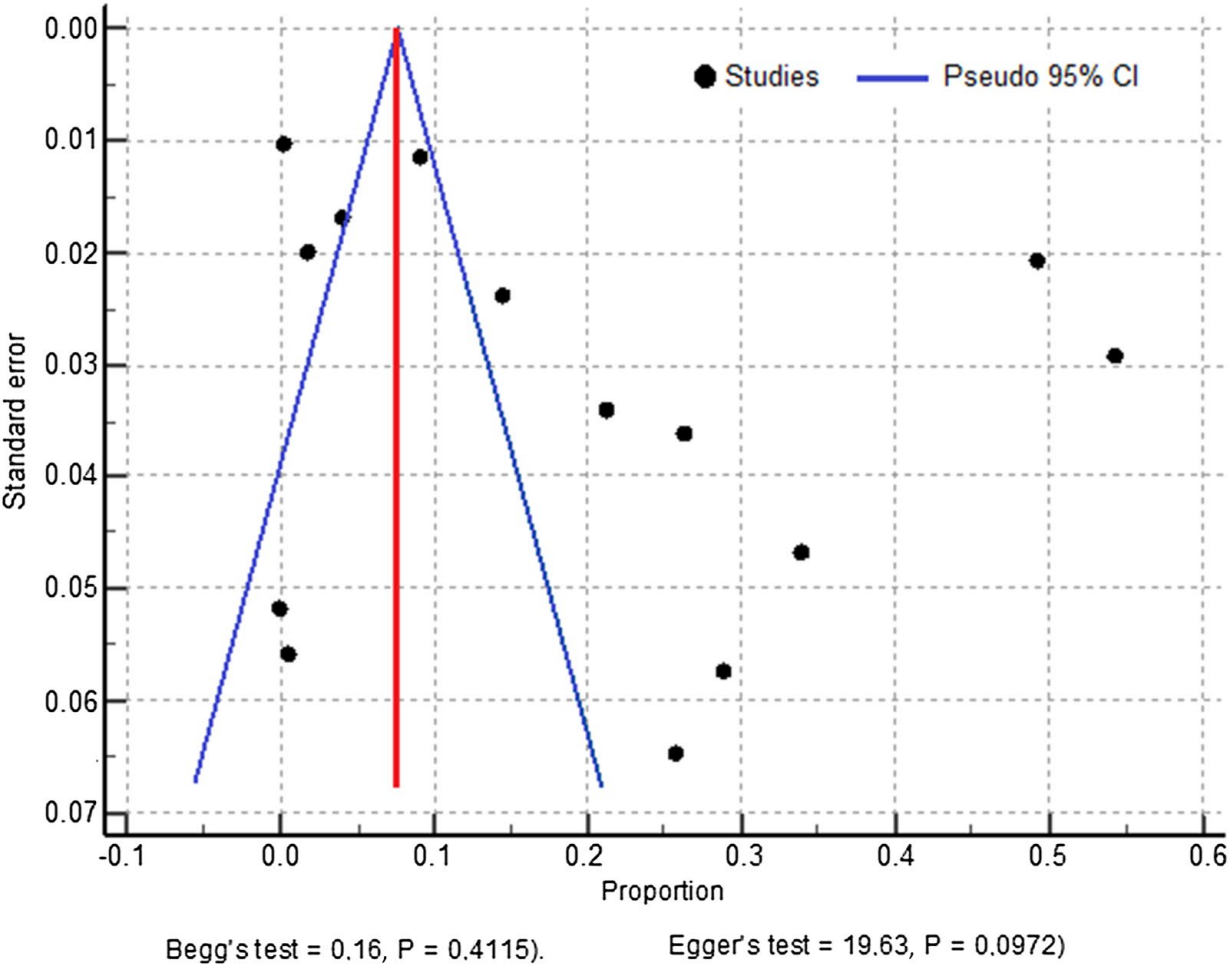
Begg’s funnel plot to assess publication bias among the included malaria prevalence studies

The presence of publication bias was evaluated subjectively using funnel plots and objectively using the Egger’s test and Begg’s test. The studies’ effect sizes were plotted against their standard errors to generate the corresponding funnel plot (Fig. 6). Visual evaluation of the obtained funnel plot showed an asymmetrical picture. However, due to its subjectivity in evaluating the presence of publication bias, Egger’s test and Begg’s test were performed. Results showed no statistical evidence of publication bias using these two tests (Egger's test, coefficient = 19.63, 95% CI: -4.1441 to 43.4050, *P* = 0.0972 and Begg's test, Kendall's Tau = 0.16, *P* = 0.4115).

## Discussion

The present systematic review and meta-analysis were carried out to determine malaria prevalence and *Plasmodium* species using data from 16 eligible prevalence studies involving 40,138 participants from 18 study sites in Mauritania. The overall prevalence of malaria was 14.9%, 24.3%, and 25.6% using microscopy, RDT, and PCR, respectively. Although PCR consistently showed a higher sensitivity in detecting malaria cases than microscopy and RDT, the low performance of routine microscopy observed in this meta-analysis may be due to several factors, including lack of skilled and competent laboratory technicians in many health facilities which negatively impacted the quality of microscopic diagnosis of malaria as previously reported [35, 36]. Nevertheless, the proportions of malaria positivity obtained in the present study is comparable to the pooled estimate of malaria prevalence in Mali (13% in general population and 19% in children younger than 5 years old) [37, 38], but higher than the proportional malaria morbidity reported from Senegal (3.3% in general population) [39]. On the other hand, the overall pooled prevalence of *P. falciparum* and *P. vivax* infections were 51.1% and 37.6%, respectively. The high proportion of *P. vivax* observed in Mauritania is unique in West Africa, where *P. falciparum* is widespread and predominant. Elsewhere in Africa, *P. vivax* is also present at a relatively high prevalence rate in the Horn of Africa and Madagascar [40]. The findings reported here calls for further revision of the global distribution map of *P. vivax* malaria, notably in Africa, which should recognize Mauritania as a *P. vivax* endemic country.

Indeed, the epidemiological situation in Mauritania is somewhat comparable to that of the Horn of Africa, particularly in Ethiopia, where the ratio of *P. falciparum* and *P. vivax* is about 3:2 [41]. Moreover, a high heterogeneity of malaria prevalence was observed in this systematic review and meta-analysis, suggesting variable and unstable seasonal malaria transmission in the country [1–3, 21, 27].

According to the subgroup analysis, the pooled prevalence of malaria was found to be very low (1%) among asymptomatic subjects compared to symptomatic subjects (21.6%). The difference in malaria prevalence between the two sub-groups is likely due to the low level of parasite density commonly observed among asymptomatic subjects, making them mostly undetectable by microscopic examination of blood smears and RDT for malaria [42]. In addition, studies suggested that low parasitaemia characterizes malaria infections in Mauritania, particularly for *P. vivax* infections, which increases the risk of misdiagnosis by microscopy and RDT [3, 6, 20].

Asymptomatic subjects rarely seek medical treatment. Therefore, they serve as an important parasite reservoir by maintaining parasite transmission [43]. Asymptomatic carriers have been identified as the major source of gametocytes, contributing to the persistence of malaria transmission [44]. Moreover, in low endemic areas, as in certain regions in India, it was reported that a small percentage of asymptomatic carriers is sufficient for malaria resurgence [45].

There are several limitations in the present study. Although only symptomatic/asymptomatic status of the enrolled participants has shown a significant contribution to the observed heterogeneity among studies, numerous other factors can explain the observed heterogeneity. For instance, differences in methodology, the characteristics of study participants, the existing variation in the environmental conditions, such as rainfall, residents’ lifestyles, and knowledge and practice of the study participants towards malaria, are potentially important factors, but many of these variables were not addressed in the studies included in the present review to allow further investigation. One of the most likely reasons for this variation could be that some of the studies were conducted during the years with unusually high amount of rainfall, such as in 2013 in Nouakchott (130 mm in one month, as compared to the average of 50 mm in other years) [23] and in 2015 in Kobeni, southeastern Mauritania (450 mm in three months, compared to the mean of 300 mm in other years) [3]. The study also revealed that malaria prevalence in the Sahelian zone (11.3%) was significantly lower than that observed in the Saharan zone (21.8%). This is most likely due to the high number of asymptomatic subjects enrolled during the studies carried out in the Sahelian zone (12,314/22,034; 55.9%), compared to the number of asymptomatic carriers included in the Saharan zone (1,707/8,963; 19%). As the overall malaria prevalence in asymptomatic subjects observed across studies is very low (1%), the high proportion of asymptomatic individuals could explain the low malaria prevalence in such studies.

In addition, the present study revealed a discrepancy between microscopy and PCR in detecting malaria parasites. While microscopy detected four human malaria species commonly found in Africa (i.e., *P. falciparum*, *P. vivax*, *P. ovale*, and *P. malariae*), PCR only detected three of them (i.e. all except *P. ovale*). Even among experienced microscopists, parasite misdiagnosis could occur due the morphological similarity between blood stages of *P. vivax* and *P. ovale* [46]. For instance, in the study conducted by Mint Lekweiry et al. [19], microscopic diagnosis revealed 15 positive slides with *P. ovale*. However, PCR performed on these samples showed that these cases were actually *P. vivax* [4]. This finding suggests that, for research purposes, PCR should probably be the gold standard to confirm malaria diagnosis. Other limitations include the assumption that interventions have had a minimal effect on malaria prevalence during the period from 2000 to 2023 in Mauritania. Analysis of data collected in Nouakchott tends to support the argument that, more than strategic interventions, climatic and environmental changes may account for changing malaria epidemiology, at least in the capital city [6]. Despite these limitations, the overall malaria prevalence during the study period may be considered as a robust estimate against which the impact of current and future interventions can be measured for evaluation of the effectiveness of interventions. It would be important to compare malaria positivity rates according to *Plasmodium* species (*P. falciparum* versus *P. vivax*), geographic zones (Sahelian versus Saharan), and study population (symptomatic patients versus asymptomatic carriers).

The results of this study have repercussion on Mauritanian malaria elimination programme [15]. First, malaria elimination strategy should take into consideration the distribution of malaria parasites in the country as revealed by the present meta-analysis. Indeed, at present, *P. falciparum* and *P. vivax* seem to evolve in distinct geographical zones with minimal interaction, as evidenced by low proportions of mixed *P. falciparum-P. vivax* infections revealed by PCR, with *P. vivax* in the Saharan zone and *P. falciparum* in the Sahelian zone. However, this situation has not been taken into consideration, and malaria control strategy in Mauritania has been designed to target only *P. falciparum* in the entire country [4]. Secondly, studies on knowledge, attitude and practices (KAP studies) towards malaria in the general population in Mauritania are required. KAP studies are one of the main components in malaria control strategies and a common tool that can capture critical information to guide the design of control interventions and ensure community involvement, acceptance, and adherence [39]. Furthermore, assessment of submicroscopic malaria infection in the asymptomatic population is critical for the success of malaria control programme. Indeed, asymptomatic malaria infections represent a major challenge toward malaria elimination.

## Conclusion

The present meta-analysis revealed that *P. falciparum* and *P. vivax* are widespread in Mauritania. This epidemiological situation implies that distinct intervention measures, including accurate parasite-based diagnosis and appropriate treatment of confirmed malaria cases, are critical for a successful malaria control and elimination programme in Mauritania [47].

## Supplementary Information

**Additional file 1: Table S1.** Summary of search keywords/terms.

**Additional file 2: Table S2.** Excluded studies reporting malaria prevalence in Mauritania and reasons for their exclusion.

## Data Availability

All data generated or analysed during this study are included in this published article and its supplementary information files.

## Abbreviations

ACT: Artemisinin-based combination therapy
CI: 95% Confidence intervals
HRP2: Histidine-rich protein-2
I^2^: Inconsistency index
IPTp-SP: Intermittent preventive treatment in pregnancy with sulfadoxine-pyrimethamine
IRD: Institut de Recherche pour le Développement (the French National Research Institute for Sustainable Development)
JBI: Joanna Briggs Institute
KAP: Knowledge, attitude and practices
LLIN: Long-lasting insecticidal nets
PCR: Polymerase chain reaction
pLDH: Plasmodial lactate dehydrogenase
PRISMA: Preferred Reporting Items for Systematic Reviews and Meta-Analyses
RDT: Rapid diagnostic test
SMC: Seasonal malaria chemoprevention
WHO: World Health Organization
