# Supplementary material for: Malaria prevalence in Mauritania: a systematic review and meta-analysis
Source: Malar J. 2023 May 2;22:146. doi: 10.1186/s12936-023-04569-4 (PMC10152621; doi:10.1186/s12936-023-04569-4)
Supplement: Supplementary file 1 — Additional file 1: Table S1. Summary of search keywords/terms. [file 12936_2023_4569_MOESM1_ESM.docx]

**Additional file 2: Table S2. Excluded studies reporting malaria prevalence in Mauritania and reasons for their exclusion**

| **Study number** | **Authors** | **Title** | **Year** | **Journal** | **Reason for exclusion** |
| --- | --- | --- | --- | --- | --- |
| 1 | Ba et al. | Epidemiology of malaria in the city of Kaedi (Mauritania, 2014) | 2018 | Tunisie Médicale | Malaria prevalence data are the same as in Gbalegba *et al*. [26] |
| 2 | Ba et al. | [Seasonal transmission of malaria in the Senegal River Valley: case study of the city of Kaedi-Mauritanie] | 2019 | Pan African Medical Journal | Malaria prevalence data is the same as in Gbalegba *et al*. [26] |
| 3 | Boushab et al. | Clinical features and mortality associated with severe malaria in adults in southern Mauritania | 2021 | Tropical Medicine and Infectious Diseases | *P. falciparum* malaria severity |
| 4 | Eberl et al. | Prevalence of polymorphisms in the dihydrofolate reductase and dihydropteroate synthetase genes of *Plasmodium falciparum* isolates from southern Mauritania | 2001 | Tropical Medicine and International Health | Weakness of the methodology with regard to the purpose of the present meta-analysis |
| 5 | Jordan et al. | Population structure of *Plasmodium falciparum* isolates during an epidemic in southern Mauritania | 2001 | Tropical Medicine and International Health | Weakness of the methodology with regard to the purpose of the present meta-analysis |
| 6 | Wurtz et al. | Vivax malaria in Mauritania includes infection of a Duffy-negative individual | 2011 | Malaria Journal | Malaria prevalence data is the same as in Lekweiry *et al*. [19] |
| 7 | Lekweiry et al. | Molecular surveillance of drug-resistant *Plasmodium vivax* using pvdhfr, pvdhps and pvmdr1 markers in Nouakchott, Mauritania | 2012 | Journal of Antimicrobial Chemotherapy | Malaria prevalence data is the same as in Lekweiry *et al*. [19] and Lekweiry *et al*. [20] |
| 8 | Lekweiry et al. | Malaria in Mauritania: retrospective and prospective overview | 2015 | Malaria Journal | A literature overview |
