# Supplementary material for: Malaria prevalence in Mauritania: a systematic review and meta-analysis
Source: Malar J. 2023 May 2;22:146. doi: 10.1186/s12936-023-04569-4 (PMC10152621; doi:10.1186/s12936-023-04569-4)
Supplement: Supplementary file 2 — Additional file 2: Table S2. Excluded studies reporting malaria prevalence in Mauritania and reasons for their exclusion. [file 12936_2023_4569_MOESM2_ESM.docx]

**Additional file 1: Table S1. Summary of search keywords/terms**

| **Database** | **Search strategy** |
| --- | --- |
| Web of Science (Core Collection)  n = 64 | TS=(Plasmodium OR malaria*) AND (TS=Mauritania* OR CU=Mauritania) AND DOP=2000-01-01/2023-01-20 |
| PubMed  n = 62 | (Plasmodium[Title/Abstract] OR malaria*[Title/Abstract] OR "Malaria"[MeSH Terms]) AND (Mauritania*[Title/Abstract] OR Mauritania*[Affiliation] OR "Mauritania"[MeSH Terms]) AND ("2000/01/01"[Date – publication]: "2023/01/20"[Date – publication]) |
| Scopus  n = 86 | TITLE-ABS-KEY(Plasmodium OR malaria*) AND (TITLE-ABS-KEY(Mauritania*) OR AFFILCOUNTRY(Mauritania)) AND PUBYEAR AFT 1999 AND PUBYEAR BEF 2023 |
| French National Research Institute for Sustainable Development (IRD) database  n = 8 | Manually searched |
